# Supplementary material for: Exploring Disparities in Maternal Residential Proximity to Unconventional Gas Development in the Barnett Shale in North Texas
Source: Int J Environ Res Public Health. 2019 Jan 23;16(3):298. doi: 10.3390/ijerph16030298 (PMC6388132; doi:10.3390/ijerph16030298)
Supplement: Supplementary file 1 [file ijerph-16-00298-s001.pdf]

International Journal of
Environmental Research and Public Health

MDPI

Supplementary Table 1. Multivariable associations between sociodemographic variables and the count of active UGD wells within 0.8 km of residential address among 164,658 women with a singleton birth in the 24-county Barnett Shale area, Texas, 2010-2012, stratified by rurality.

	**Urban[1,2] (n = 143,766)**		**Rural (n = 20,889)**	
	**n**	**Count Ratio (95% CI)**	**n**	**Count Ratio (95% CI)**
Maternal age (years)				
≤ 20	21,025	1.0 (ref)	3,749	1.0 (ref)
21-25	34,969	1.08 (1.01, 1.16)	6,390	1.03 (0.91, 1.18)
26-30	41,034	1.12 (1.05, 1.20)	5,876	1.11 (0.97, 0.28)
31-35	31,540	1.01 (0.97, 1.09)	3,378	1.29 (1.10, 1.51)
36+	15,200	1.01 (0.93, 1.10)	1,497	1.12 (0.92, 1.37)
Maternal education				
College graduate	35,462	1.0 (ref)	3,654	1.0 (ref)
Some college	33,896	1.34 (1.26, 1.43)	6,896	0.97 (0.85, 1.11)
High school graduate	44,165	1.12 (1.05, 1.19)	6,195	1.06 (0.91, 1.22)
Less than high school	30,166	1.39 (1.29, 1.50)	4,103	0.94 (0.80, 1.11)
Maternal race/ethnicity				
Non-Hispanic White	47,568	1.0 (ref)	14,208	1.0 (ref)
Hispanic	60,243	0.65 (0.61, 0.68)	5,168	0.94 (0.84, 1.04)
Non-Hispanic Black	25,389	0.83 (0.78, 0.89)	1,011	0.61 (0.48, 0.76)
Other	10,568	0.75 (0.69, 0.79)	503	0.89 (0.66, 1.19)
Adequacy of prenatal care				
Adequate	6,266	1.0 (ref)	1,216	1.0 (ref)
Inadequate	31,564	1.17 (1.11, 1.24)	3,592	1.20 (1.06, 1.36)
Intermediate	21,381	0.99 (0.11, 1.05)	2,956	1.15 (1.01, 1.32)
Adequate plus	58,906	0.86 (8.11, 0.92)	8,888	0.87 (0.77, 0.98)
Unknown	25,651	0.61 (6.11, 0.68)	4,238	0.97 (0.80, 1.19)
ICE Race/ethnicity and income[3,4]				
Tertile 3 (high)	44,548	1.0 (ref)	9,915	1.0 (ref)
Tertile 2	46,186	0.66 (0.63, 0.70)	9,781	0.61 (0.75, 0.67)
Tertile 1 (low)	53,032	0.75 (0.71, 0.79)	1,193	0.48 (0.39, 0.60)

CI = confidence interval; ref = reference category; ICE = Index of Concentration at the Extremes; [1] Model included maternal age (years), maternal education, maternal race/ethnicity, prenatal care utilization index, and ICE race/ethnicity and income; [2] Census tracts classified as urban if located within Dallas, Denton, or Tarrant counties; [3] n=3 missing; [4] ICE categorized into tertiles for stratified analysis due to small sample sizes in rural subsample, cutpoints (-1 = extreme concentration of deprivation; 1 = extreme concentration of privilege) tertile 1: 0 to <-0.116, tertile 2: -0.116 to <0.106, tertile 3: 0.106 to 1.

© 2019 by the authors. Submitted for possible open access publication under the terms and conditions of the Creative Commons Attribution (CC BY) license (http://creativecommons.org/licenses/by/4.0/).
